# American Dental Association

**Published:** 1887-10

**Authors:** 


### AMERICAN DENTAL ASSOCIATION.

### TWENTY-SEVENTH ANNUAL MEETING.

Reported for the Independent Practitioner.

Continued From Page 487.

### WEDNESDAY MORNING SESSION.

Section III, "Operative Dentistry," was called. The report was presented by the Chairman, Dr. E. T. Darby. No papers were offered by members of the Section, but a number of topics for discussion were suggested.

The report stated that a year had passed since the presentation before this body of the Herbst method of filling teeth. Has the experience of the year demonstrated that as a system it is destined to supersede the old methods? Its introduction has stimulated us in various ways. The introduction of the Wolrab gold has resulted in the production of better gold here. The debates upon the rotation method have brought about a better comprehension of the principles involved in filling teeth, and have made us acquainted with new possibilities, and have thus resulted in great good, even if not one dentist should adopt the system exclusively.

The system of implantation of teeth, as practiced by Dr. W. J. Younger, has startled the whole profession. That many of his cases have resulted in at least a temporary success, no one will dispute. But it requires time to prove the entire reliability of the practice, and it is now too soon to pronounce positively concerning its merits. The possible danger would seem to lie in the fact that changes in the territory surrounding the newly implanted tooth may come with time.

The various kinds of separators lately introduced were referred to, and their relative merits discussed.

In operative dentistry, the advance during the year has been in methods. No specially new principles, aside from those referred to, have been enunciated, but there has been a steady improvement in intelligence and comprehension of basal laws. What is most needed now is a plastic material which shall meet all the requirements of a perfect permanent filling. Amalgam has certain defects, and the other plastics are, at best, but temporary makeshifts. There must be in nature the long looked-for plastic. Could our funds be devoted to a better purpose than the stimulation of chemists to the production of the desideratum ?

No perfect obtunder for sensitive dentine has yet been presented. Absolute dryness and a temperature of from 90° to 100°, obtained by means of a stream of hot air, seem to be the most effectual means yet devised. What is now needed is an apparatus which shall easily and effectively heat the air to the proper temperature, and deliver it in the cavity in a satisfactory manner.

DISCUSSION.

*Dr. Morrison*—To accomplish the carrying down of the rubber-dam to the cervical margin of the teeth, I stumbled upon a device which has proven most effectual. I take a strip of elastic rubber, of proper length and thickness, and stretching it between the fingers until it will pass between the teeth, I force the dam to the point where it should rest. It will not cut the dam or tear it, while it will carry it to place much better than anything I have tried.

For many years I have been working upon the theory that irregularities in teeth should be prevented. Few cases would present if parents did their duty and were well informed. The deciduous teeth should be allowed to remain till pushed out by nature. In proof of his assertions, Dr. Morrison exhibited a number of casts, showing how teeth will care for themselves if permitted to remain in the mouth.

*Dr. Watkins*—Reported the case of a young miss of thirteen, whose lower anterior teeth shut entirely outside the upper ones. By wedging with cotton tape, and without the placing of any fixture in the upper jaw, the arch was so spread that the superior teeth were carried outside. Then an apparatus, consisting of an inside bar to which the teeth were attached by elastic ligatures, was placed on the lower jaw, and they were drawn into place.

*Dr. Keely*—Presented a case illustrating the evil effects of premature extraction of the second temporary molars before the eruption of the first permanent molars. It will, in nearly every case, cause a projection of the anterior teeth.

*Dr. Barrett*—Presented the case of a miss of fourteen, whose anterior teeth projected and were much shortened as if from thumb-sucking, which, however, was not the cause, the difficulty in all probability arising from the premature extraction of teeth. There were but two molars in the upper jaw, and the problem was to draw the six anterior teeth back by means of six posterior ones without danger of moving the latter. It was accomplished by carefully banding the bicuspids and molar on each side, as if for gold crowns, and then attaching these bands together by soldering to them a side plate which carried guides and a screw for drawing back a thin plate or strap, which passed across the faces of the anterior teeth, the whole apparatus being made of gold. The banding of the posterior teeth and the attachment of the bands to a side plate, presented so immovable an anchorage that tipping of the teeth was impossible.

Dr. Barrett also presented the case of a boy of seven years, whose central incisors were just appearing through the gum, all the other teeth being very short deciduous ones. By an accident, one central incisor was knocked out. Forty-four hours afterward the case presented itself. The knocked-out incisor was incomplete as to development, there being but a mere shell of the root, while the foraminal opening was almost the entire size of the root. A piece of small platinum wire was soldered at right angles to the plane of a very thin platinum plate, sufficiently large to cover the end of the root. The wire was cut to a length that admitted its introduction into the tooth-canal through the foraminal opening. The root was then filled with oxy-phosphate of zinc, and the platinum wire and plate placed in position, the latter being carefully burnished down over the end of the root. An impression in wax was taken, and a tooth about the size of the extracted one fitted in the cast and allowed to project to the length to which the original would probably have grown had development continued. Very thin platinum was then carefully burnished over the plaster casts of the teeth, it was stiffened by flowing gold over it, filled with oxy-phosphate of zinc, the extracted tooth having been first placed in position and the teeth all carefully dried, it was carried to place and held there until the plastic material had set. In three weeks reunion was sufficiently complete, and now, after one year, the extracted tooth cannot be identified by a stranger.

*Dr. Priest*—Forty years ago it was considered a great feat to accomplish the moving of any of the natural teeth. To-day it is but a simple matter.

*Dr. H. A. Smith*—When a tooth is to be moved, if a portion of the alveolar plate opposite it, either inside or outside, according to the direction in which it is to be moved, be cut away, it will greatly facilitate the process.

*Dr. Knapp*—The regulation of teeth is the most vexatious business a dentist engages in. A part of the pay, at least, should be demanded in advance. If this be not done the patient is apt to get discouraged and stop half way, placing the blame upon the dentist and defrauding him of the fees already earned.

Subject passed.

(TO BE CONTINUED.)

